# A Case Report on Collagen Nevus: A Rare Clinical Entity

**DOI:** 10.7759/cureus.81991

**Published:** 2025-04-10

**Authors:** Venkata Raghavendra Chintha, Manimaran R, Koppolu Kanchana, S Balakrishnan, Karthikeyan Selvaraj

**Affiliations:** 1 General Surgery, Sree Balaji Medical College and Hospital, Chennai, IND; 2 Plastic Surgery, Sree Balaji Medical College and Hospital, Chennai, IND

**Keywords:** collagen, collagen nevi, collagenomas, eruptive collagenoma, hamartoma, skin-colored lesions

## Abstract

Collagen nevi, or hamartomas, are abnormal collagen growths in the skin. These lesions have a varied presentation. Mostly, these lesions are harmless, usually associated with cosmetological concerns. This is the case of a 20-year-old male patient with multiple skin-colored lesions on his back since the age of 10 years. The patient was managed by surgical excision of the lesions. He was followed up at three months post-procedure, which showed the patient recovering well with no recurrences.

## Introduction

Abnormal accumulation of the collagen under the skin results in a condition termed "collagen nevus". Resultant lesions are also termed "hamartoma". It is caused majorly due to the changes in the extracellular matrix. These changes result from an increase or changes in the structural constituents of this matrix [1]. Collagen nevi are benign in nature, usually formed by malformations and disorganization of skin tissue. The three main components constituting a connective tissue nevus are collagen, elastin, and proteoglycans as a single constituent or in combination [2,3]. It is characterized by the whitish or skin-colored nodule-like overgrowths under the dermis with a size ranging between 1 mm and 20 mm. The incidence has been noted in cases as young as infants to >70 years of age, equally affecting both males and females [4,5]. There is a dearth of published case reports of hamartomas or collagen nevi in the Indian population; hence, this case of a 20-year-old male patient is reported, who presented with multiple swellings for the last 10 years. The swellings were not associated with any pain. The patient was managed by excision biopsy and was discharged the same day.

## Case presentation

**Figure 1:**
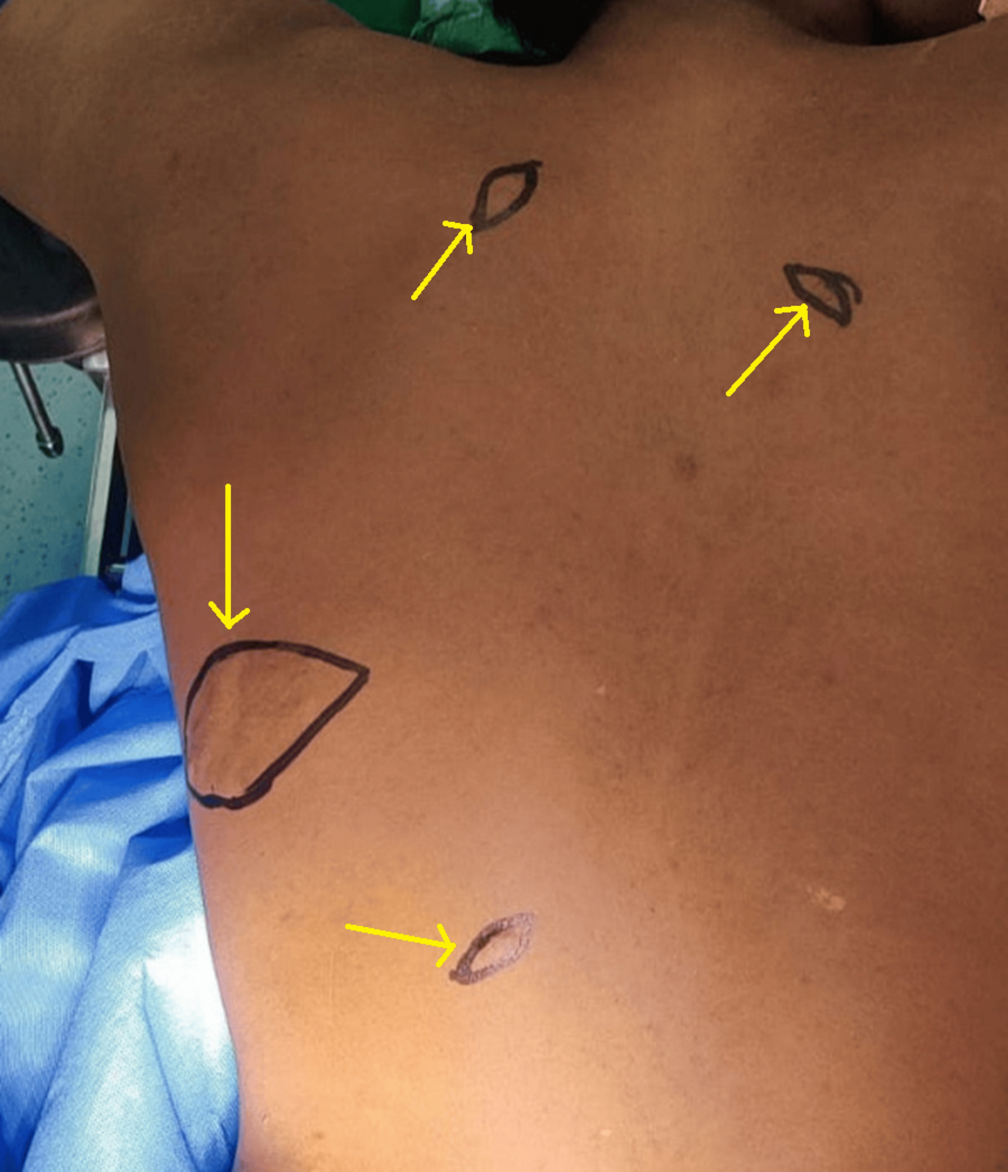
Physical presentation of the swellings on the patient's back

A 20-year-old male patient presented to the outpatient department with a major complaint of multiple painless nodule-like swellings on his back. The swellings appeared first when he was 10 years of age. The patient was a non-alcoholic and a non-smoker. There was no significant family or medical history. Also, there was no history of trauma, a sudden increase in size, or fever. Physical examination showed multiple ill-defined raised lesions observed over his back, which were non-tender, immobile, and firm. The swellings were skin-colored and insidious in onset and gradually progressive. It was also not associated with any pain or any kind of discharge (Figure 1).

**Figure 2:**
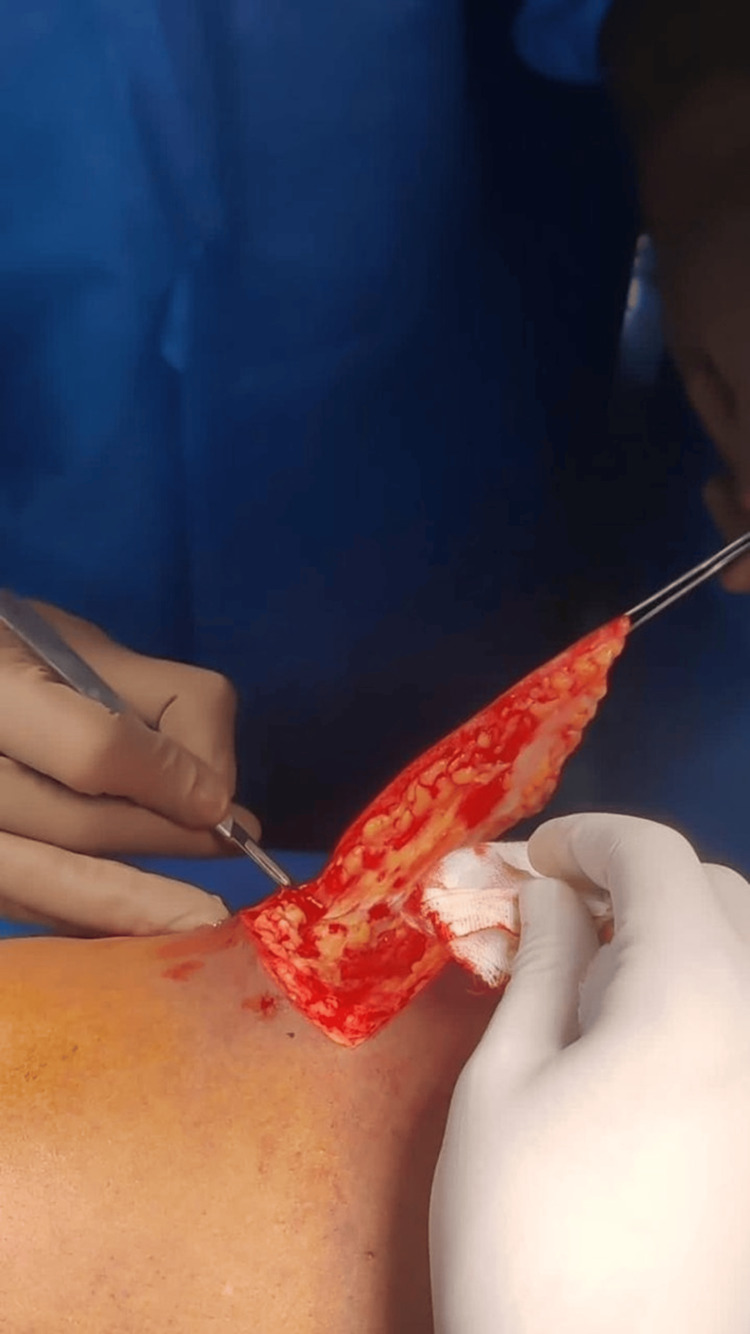
Intraoperative image of the patient

**Figure 3:**
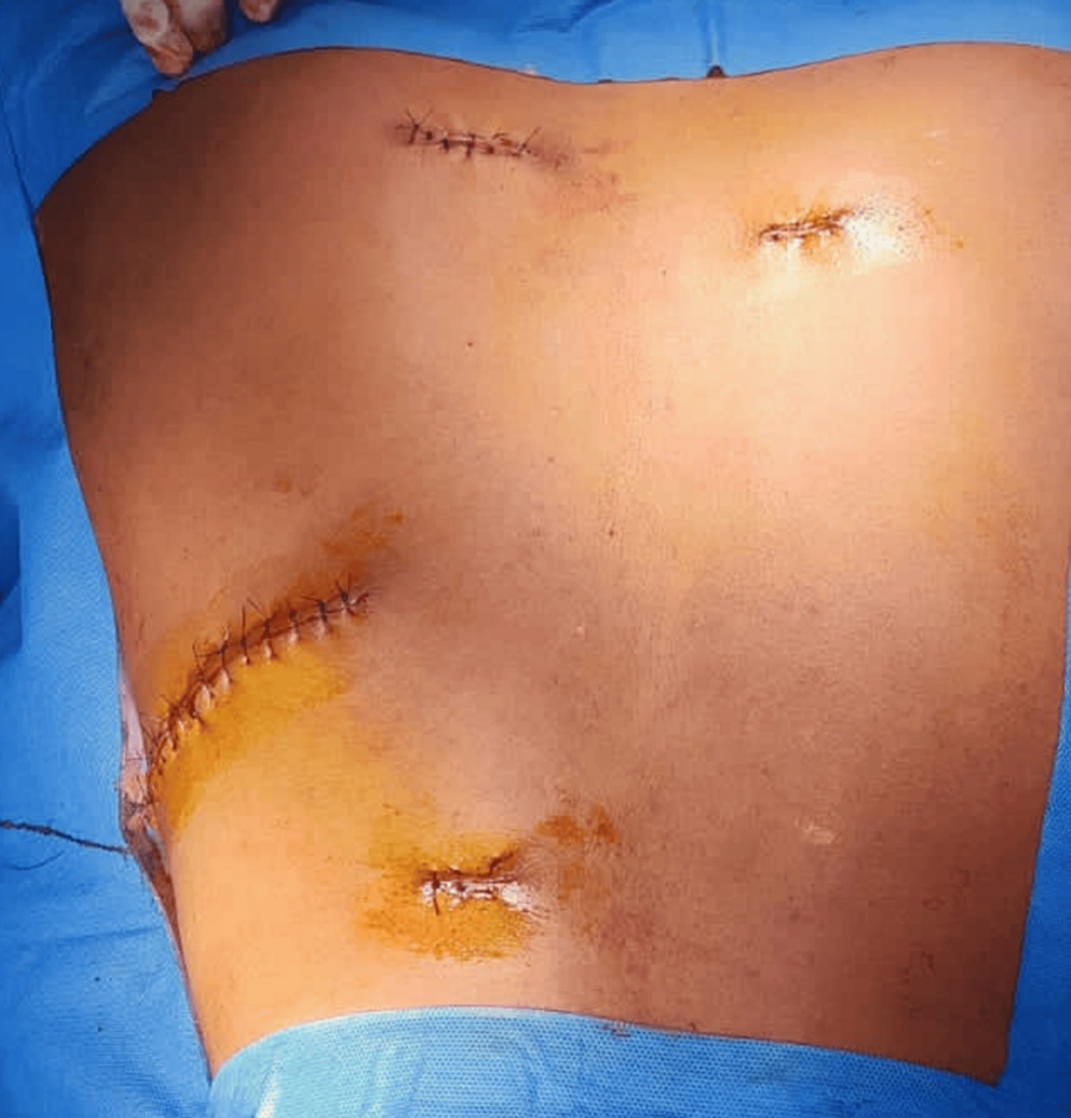
Postoperative presentation

The patient was diagnosed, based on clinical examination, with collagen nevus. He has requested for the excision of lesions due to cosmetic purposes as he was planning to go abroad, for which he was treated with. The lesions were excised with a 2-3-mm margin clearance. The procedure was uneventful (Figure 2 and Figure 3).

**Figure 4:**
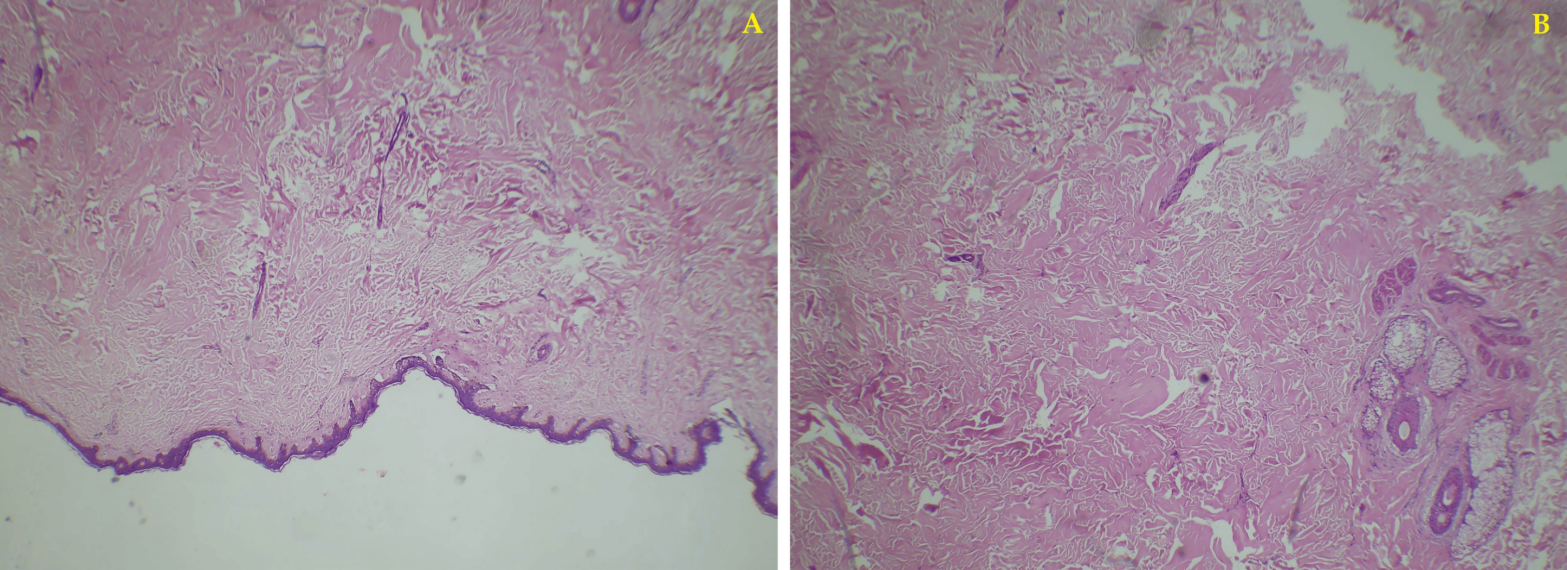
Hematoxylin and eosin stain slide showing mild hyperkeratosis and flattening of rete ridges: (A) 10× magnification and (B) 40× magnification

**Figure 5:**
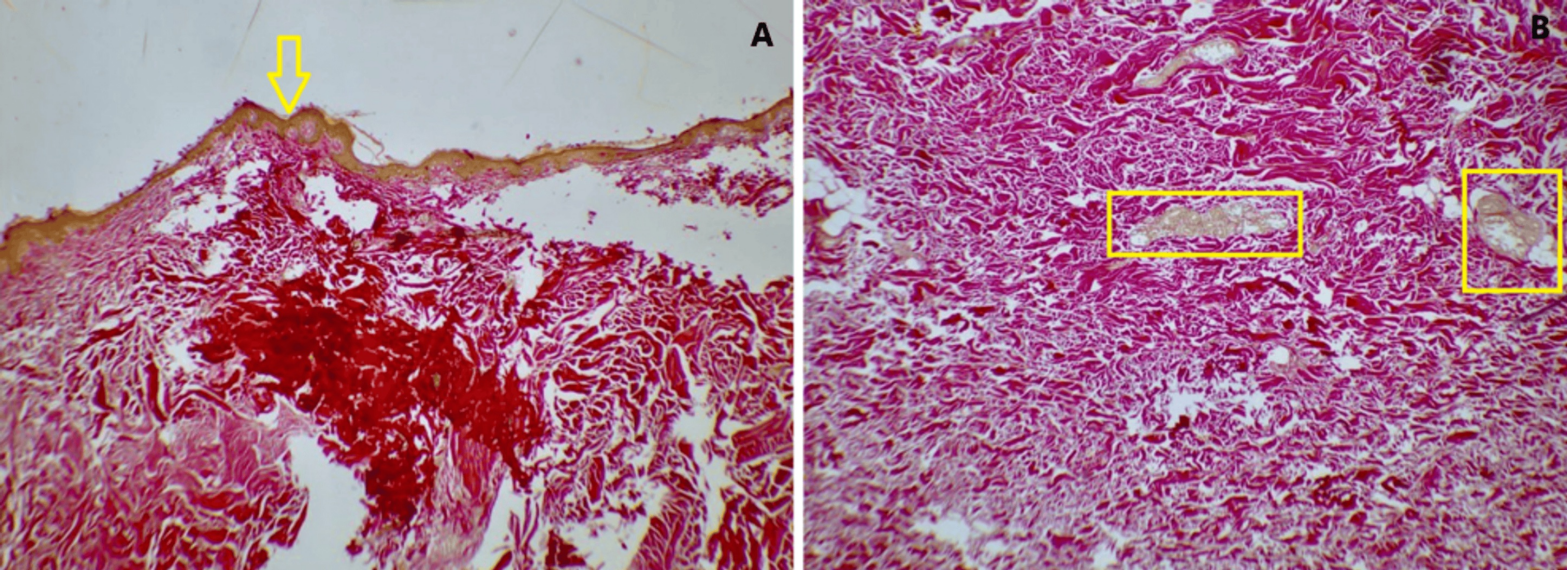
Verhoeff-Van Gieson stain showing dermal collagenization: (A) 10× magnification and (B) 40× magnification

The excised materials were sent for histopathological analysis. The histological findings of the hematoxylin and eosin staining showed squamous epithelium with mild hyperkeratosis and flattening of rete ridges in patches. The upper dermis showed collagen bands with few attenuated adnexal structures and occasional hair follicles which was suggestive of collagen nevi or hamartoma. This was further supported by the Verhoeff-Van Gieson stain which showed dermal collagenization (Figure 4 and Figure 5).

The patient was prescribed tablet amoxicillin (500 mg)+clavulanic acid (125 mg), tablet domperidone (30 mg)+pantoprazole (40 mg), tablet trypsin chymotrypsin (100000 AU) for five days, tablet aceclofenac for three days, and multivitamins for seven days. The patient was followed up twice at two-week intervals till one month followed by one monthly follow-up till three months.

## Discussion

Hamartomas arise due to the imbalance of collagen, elastin, and proteoglycans. The lesions with predominant collagen and elastin are termed as collagenoma and elastoma, respectively [6]. The nevi may also include excess glycosaminoglycans (the ground substance of the dermis), excess smooth muscle, or excess fat. Collagen nevi are rare and benign skin lesions, which are observed typically in early childhood [5], but there are cases reported in the geriatric population as well [2,4]. The literature sometimes mentions the occurrence of collagen nevi in conjunction with certain syndromes, such as syndromic nevus and multiple collagen nevi. These associations highlight that, in some cases, multiple collagen nevi may occur as part of a clinical syndrome, but this remains a rare occurrence. These lesions usually appear in multiple numbers and can be indicative of different underlying pathologies, such as Buschke-Ollendorff syndrome, tuberous sclerosis, and familial or eruptive cutaneous collagenoma [3,7]. It has been classified into different categories based on its genetic, histopathological, and clinical traits. It has a specific clinical appearance, mostly benign on appearance, and specific distribution patterns with specific histological features such as increased collagen with decreased elasticity [1,8]. 

Based on histological features, it might be a classic collagen nevus (excess of collagen fibers in the dermis), spindle cell collagen nevus (spindle-shaped cells in addition to the collagen fibers), palisaded collagen nevus (fibers are arranged in a palisade), or fibrous collagen nevus (predominantly fibrous tissue). Eruptive collagenoma has been reported in the trunk and lower extremities of the body. This case also observed the lesions on the back of the patient since the age of 10 years [2,4,5,9]. However, these lesions have been associated with mutations in tumor suppressor genes (TSC1 and TSC 2) [9]. There are no standard therapeutic options available for the same as most of the lesions are left untreated due to their asymptomatic nature. Though symptomatic treatments are available, most of them target aesthetics [6,10]. This case was managed by surgical excision, followed by a histopathological analysis of the excised specimen. Histopathological analysis of the tissue biopsy is commonly carried out in most cases to confirm the diagnosis of collagen nevi or collagenoma [4-6,11]. After excision, recurrence of collagen nevi is rare but possible, and the literature suggests that close follow-up may be necessary to ensure complete removal. There are instances where the lesion may appear to grow or change over time, but these changes are typically benign and not indicative of malignancy. As there is a paucity of published clinical data, more cases need to be reported to develop clinical evidence-based therapeutics that can help develop standard treatment regimens.

## Conclusions

Collagen nevus is typically regarded as a benign, well-defined entity, and most cases are not associated with significant morbidity. While it can sometimes resemble other skin conditions, it is distinguishable through histopathological examination and its characteristic collagen-rich structure. The rarity of collagen nevi, combined with their benign nature, means that they are usually only studied in detail in a few case reports. Collagen nevus is typically asymptomatic and presented as multiple lesions. Attributed to its rarity, there is a need for more cases to be reported, which can aid the management procedures along with associated clinical significance.
